# Engineering of 2D Ti_3_C_2_ MXene Surface Charge and its Influence on Biological Properties

**DOI:** 10.3390/ma13102347

**Published:** 2020-05-20

**Authors:** Anita Rozmysłowska-Wojciechowska, Joanna Mitrzak, Aleksandra Szuplewska, Michał Chudy, Jarosław Woźniak, Mateusz Petrus, Tomasz Wojciechowski, Alexey S. Vasilchenko, Agnieszka M. Jastrzębska

**Affiliations:** 1 Faculty of Materials Science and Engineering, Warsaw University of Technology, Wołoska 141, 02-507 Warsaw, Poland; asiamitrzak@gmail.com (J.M.); jaroslaw.wozniak@pw.edu.pl (J.W.); Mateusz.Petrus.dokt@pw.edu.pl (M.P.); agnieszka.jastrzebska@pw.edu.pl (A.M.J.); 2 Faculty of Chemistry, Warsaw University of Technology, Noakowskiego 3, 00-664 Warsaw, Poland; aszuplewska@ch.pw.edu.pl (A.S.); chudziak@ch.pw.edu.pl (M.C.); wojciechowski16@gmail.com (T.W.); 3 Institute of Environmental and Agricultural Biology (X-BIO), Tyumen State University, 625003 Tyumen, Russia; a.s.vasilchenko@utmn.ru

**Keywords:** MXenes, delaminated Ti_3_C_2_, poly-L-lysine, antibacterial properties, cytotoxicity in vitro, mammalian cells

## Abstract

Current trends in the field of MXenes emphasize the importance of controlling their surface features for successful application in biotechnological areas. The ability to stabilize the surface properties of MXenes has been demonstrated here through surface charge engineering. It was thus determined how changing the surface charges of two-dimensional (2D) Ti_3_C_2_ MXene phase flakes using cationic polymeric poly-L-lysine (PLL) molecules affects the colloidal and biological properties of the resulting hybrid 2D nanomaterial. Electrostatic adsorption of PLL on the surface of delaminated 2D Ti_3_C_2_ flakes occurs efficiently, leads to changing an MXene’s negative surface charge toward a positive value, which can also be effectively managed through pH changes. Analysis of bioactive properties revealed additional antibacterial functionality of the developed 2D Ti_3_C_2_/PLL MXene flakes concerning *Escherichia. coli* Gram-negative bacteria cells. A reduction of two orders of magnitude of viable cells was achieved at a concentration of 200 mg L^−1^. The in vitro analysis also showed lowered toxicity in the concentration range up to 375 mg L^−1^. The presented study demonstrates a feasible approach to control surface properties of 2D Ti_3_C_2_ MXene flakes through surface charge engineering which was also verified in vitro for usage in biotechnology or nanomedicine applications.

## 1. Introduction

Since the discovery of the specific properties of graphene, there has been an avalanche like increase of interest in two-dimensional (2D) materials [1]. Progress in the field of technical sciences creates many opportunities for the development of such materials and it can now be considered that it is one of the fastest developing fields [2]. New MXene structures (i.e., light transition metal carbides, nitrides and carbonitrides) are interesting in this area because of their specific physicochemical and bioactive properties such as antibacterial properties, biosensing and excellent photothermal conversion which can be used in theranostics [3]. The first reports about them came in 2011, when a research group from Drexel University (USA) published their first work in this field [4]. In a short time, it turned out that MXenes is a very large group of new materials with anisotropic crystal structure (e.g., Ti_2_C, Ti_2_N, Nb_2_C, V_2_C, Mo_2_C, Ti_3_C_2_, Ti_3_CN, Ti_4_N_3_ or Nb_4_C_3_) with long-range in-plane ordering [5].

MXenes can be also written as ‘M_n+1_X_n_T_x_’, which reflects their stoichiometric system and surface terminations [6], where: M is a light transition metal, X—carbon and/or nitrogen, T_x_ is -OH, =O or -F chemical terminations, while n = 1, 2, 3 or 4. In the MXenes phases, there is a sandwich-type arrangement of M_n+1_X_n_ monolayers, thanks to which they are characterized by a unique flake morphology. As a result that MXene phases are developing much more dynamically in comparison to other 2D materials, apart from graphene, they are currently being considered as interesting and bringing high innovation potential in comparison with other two-dimensional materials [6].

Of all MXene phases, the best-known systems to date are those containing titanium and carbon, i.e., the Ti_3_C_2_ phase—A representative of the Ti-C system [7]. The advantage of this phase is the highest application potential among other phases and their high availability on the market which results from the well-known and developed preparation methodology. Therefore, the Ti_3_C_2_ phase has already demonstrated its usefulness and significant potential in many applications, such as energy storage [7], composite structures [8] and adsorption of organic pollutants [9]. MXenes also attracted the attention of nanomedical applications including various therapeutic techniques (diagnostic imaging, nano-systems for biosensors) [10], as well as in microbiological protection [11]. In this context, the Ti_3_C_2_ MXene phase was selected for this study as the most promising and interesting. It should be noted however that the development of 2D MXenes in nanomedicine is still at a very early stage [12]. The low availability of scientific data on their toxicity to mammalian cells [13] and bacteria [11], as well as scarce availability of simple and universal methods for controlling the composition of the surface [14,15] make it difficult to develop further. This particularly applies to the low chemical stability of the surface [14,16], the tendency to change the chemical composition, e.g., through surface oxidation, changes the stability and dispersion of these nanosystems in the physiological environment. This barrier must be overcome to confirm the assumed bioactive properties of these materials. At present, there is also not much research on their interaction with biomacromolecules [17], which also limits their practical application.

Therefore, there is no doubt that effective methods for controlling the surface of MXenes are needed. At the same time, feasible methods are desirable which not only allow to control the surface itself, but also obtain additional functionalities such as antibacterial properties, or minimization of potential cytotoxic properties [18]. A good solution can be associated with the use of organic macromolecules of biological origin [16], such as e.g., poly-L-lysine (PLL). Poly-L-lysine apart from being an antibacterial agent [19] is a particularly interesting topic because of its unique molecular system [20], which through the proper arrangement of ionic chemical groups in the polymer molecule, provides a high positive charge. It should be noted that the surface of MXenes possesses a negative surface charge [21]. It can, therefore, be assumed that through the mutual electrostatic interaction of these substances, it is possible to obtain a hybrid 2D Ti_3_C_2_/PLL system with specific functional and biological features.

Accordingly, the purpose of this work was to investigate the impact of surface modification of 2D Ti_3_C_2_ flakes with PLL on the colloidal and biological properties of the developed system. Comprehensive research was carried out, including the analysis of the morphology, structure, physicochemical properties, hydrodynamic diameter for the initial powder of 2D Ti_3_C_2_ MXene flakes as well as 2D Ti_3_C_2_ flakes surface-modified with PLL. The process of PLL adsorption was verified step-by-step by zeta potential analysis, also as a function of pH. The assumed biological features were verified in vitro using MTT cytotoxicity analysis and bactericidal testing for both pristine 2D Ti_3_C_2_ and 2D Ti_3_C_2_/PLL hybrid.

## 2. Materials and Methods

### 2.1. Preparation of the MAX Phase and 2D MXene

The Ti_3_AlC_2_ MAX phase used in the conducted analysis was obtained with the use of the powder metallurgy technique and the Spark Plasma Sintering (SPS) method. Titanium powder (GoodFellow, Wrexham, UK), aluminum powder (Benda-Lutz Skawina, Skawina, Poland) and synthetic graphite powder (Sigma Aldrich, St. Louis, MO USA) were used for this purpose. The powders were wet blended in isopropyl alcohol with a molar ratio Ti:Al:C = 3:1:1.9 using ball type mill, dried and sieved (# = 300 µm). Due to the exceptional design of graphite die it was possible to use the pressureless SPS technique [22] in the reactive synthesis process. The formula below describes the reaction which took place during the synthesis process:

(1) $$3\;\mathrm{Ti}+\mathrm{Al}+2\;C\to {\;\mathrm{Ti}}_{3}{\mathrm{AlC}}_{2}$$

It was conducted using the following parameters: temperature 1300 °C, heating rate 250 °C/min, vacuum atmosphere amounted to 5 × 10^−2^ mbar (medium vacuum). Having been cooled, the obtained MAX phase was ground using an automatic mortar grinder (Retsch KM100) and sieved (# = 300 µm). Finally, the Ti_3_AlC_2_ powder was subjected to the process of etching.

The etching is based on aluminum (Al) removal from the sandwiched -Ti_3_C_2_-Al-Ti_3_C_2_-Al-Ti_3_C_2_- structure and obtaining Ti_3_C_2_ layers interleaved by slit-shaped pores which appear in place of the removed Al atoms. The process of etching was carried out in a Teflon vessel using a hydrofluoric acid (HF) protocol. The MAX phase powder was carefully immersed in a 48% HF water solution (Sigma-Aldrich) and was continuously stirred at 250 rpm. The consequent chemical reactions result in the generation of gaseous hydrogen (H_2_). To enable mild reaction process and effective H_2_ removal, the ratio of MAX phase to HF solution was fixed at 1 g/10 cm^3^. The etching process was carried out for 24 h at room temperature with assured continuous stirring and fume hood protection. The resulting suspension was then allowed to sediment. The obtained supernatant was discarded and the Ti_3_C_2_ MXene was washed four times with deionized water and technical grade ethanol. The Ti_3_C_2_ MXene was then dried overnight at room temperature and stored at 5 °C for further use.

For the purpose of delamination, a total of 100 mg of Ti_3_C_2_ MXene powder was mixed with 10 mL of double-distilled water (DDW) containing 100 mg of tetramethylammonium hydroxide (TMAOH) in a 20 mL glass vial, and the mixture was stirred for 24 h at room temperature. The alkaline mixture (pH~10) was mildly sonicated for 6 h and washed twice via centrifugation (3500 rpm, 5 min per cycle) using 50 mL tubes to bring the pH to ~7. The stable MXene colloidal solutions were collected after 1 h of centrifugation at 3500 rpm, and the sediments were collected via vacuum-assisted filtration. The samples were then redispersed in deionized water and freeze-dried for 24 h to obtain final powdered samples.

### 2.2. Studies on Morphology and Structure

The morphology of 2D flakes of Ti_3_C_2_ MXene was examined using a scanning electron microscope (SEM, LEO 1530, Zeiss, Lake Buena Vista, FL, USA). The freeze-dried Ti_3_C_2_ MXene flakes were directly placed onto a sticky carbon tape. The sample was then sputtered with a thin carbon layer (SCD 005, BAL-TEC, now Leica Microsystems GmbH, Wetzlar, Germany). The SEM analysis was performed at an accelerating voltage of 5.0 kV. The morphology of the 2D flakes of the Ti_3_C_2_ MXene phase was also studied using a transmission electron microscope (TEM, PHILIPS CM 20, Philips International B.V., Amsterdam, The Netherlands). The sample in the form of an aqueous dispersion (before freeze-drying) was placed dropwise on a copper mesh covered with carbon film. The layered structure of the petals was examined on the cross-section at atomic resolution, using Fourier transformation (FFT) together with the subsequent inverted Fourier transformation (IFFT). An intensity analysis was also performed on a line perpendicular to the plane formed by the individual 2D flakes. This made it possible to analyze the distances between the lightest periods of the structure (bright stripes). The elemental composition of 2D flakes was also checked using an Energy Dispersive X-Ray Spectroscopy (EDS, Philips International B.V., Amsterdam, The Netherlands) coupled with a transmission electron microscope PHILIPS CM 20.

### 2.3. Studies on Physical Properties

Analysis of the physical properties of 2D flakes of the Ti_3_C_2_ MXene phase was performed using the nitrogen physical sorption isotherm, which refers to the volume of adsorbed gaseous N_2_ as a function of N_2_ relative pressure. Measurement of the nitrogen physical sorption isotherm was conducted experimentally using the Quadrasorb-SI device (Quantachrome Instruments, Boynton Beach, FL, USA). Prior to measurement, the freeze-dried sample was degassed in vacuum at 150 °C for 24 h to both enable efficient surface leaning and minimize the adverse surface oxidation of MXene. Measurements of N_2_ sorption on the surface of the 2D Ti_3_C_2_ MXene phase studied were recorded for the full relative pressure range (p/p° from 0 to 1) at −195.8 °C. The specific surface area of the 2D Ti_3_C_2_ MXene was obtained by the Brunauer, Emmett and Teller (BET) method in the relative pressure range p/p° of 0.05–0.35. Pore shape analysis was performed by comparing the experimentally determined isotherm obtained for 2D flakes of the Ti_3_C_2_ MXene phase, together with four physical gas sorption isotherms classified by the International Union of Pure and Applied Chemistry (IUPAC).

### 2.4. Studies on Colloidal Properties and PLL Adsorption

For the preparation of the initial suspension, 0.50 ± 0.01 mg of freeze-dried 2D Ti_3_C_2_ MXene was suspended in 1.000 ± 0.001 mL distilled water, to obtain a concentration of 5 × 10^2^ mg L^−1^. The prepared solution was subjected to ultrasound (30 s, 1 s work/1 s break i.e., 15 s of total working time and 15 s of total resting time). The initial solution was then diluted to a concentration of 5 × 10^−5^ mg L^−1^ for modification with PLL in different weight ratios of MXene to PLL (from 1:0 to 1:10) whereas 1:1 was assessed experimentally as the optimal ratio. Three measurement series were conducted at 25 °C. This method was also used to measure the zeta potential of 2D Ti_3_C_2_ modified with PLL. The concentrations of the analyzed colloidal solutions were maintained the same for all further studies.

Zeta potential and hydrodynamic diameter measurements (DLS) were analyzed using the NANO ZS ZEN3500 analyzer (Malvern Instruments, Malvern, UK) and the DTS1060 measuring cell was used for sample testing. The analyzer was equipped with an MPT-2 automatic titrator and degasser. The device was operated at 25 °C during measurements. Smoluchowski’s model was used to study the zeta potential, while the particle size measurement was performed using the dynamic light scattering (DLS) technique. The following device parameters were set: measurements of MXene flake sizes: 20 repetitions for each measurement, duration of each unit measurement: 1 s, measurements of the zeta potential: 30 repetitions for the measurement. Measurements were also made as a function of pH. The MXene suspension was titrated with 0.1 M NaOH and 0.1 M HCl solutions. Zeta potential and particle size measurements were carried out in the pH range from 4 to 11 in steps of 0.5 pH unit. Particle size distributions were obtained for three solutions of MXenes in a DDW. Quantitative data from the detector (so-called distribution by quantity) was used to analyze the flake size. The distribution by quantity allows plotting signals from individual flakes. However, quantitative data derived from the intensity of the recorded signals (so-called distribution by intensity) was used for the analysis of larger floccules. This is because agglomerates are larger than particles and by scattering laser light, they give significantly higher intensity values. This relationship was used to analytically distinguish these two types of signals and hence the two fractions present in the material (i.e., flakes and agglomerates).

For the adsorption of PLL on the surface of MXene, poly-L-lysine hydrobromide (P2636, Sigma-Aldrich, CAS: 25988-63-0, MDL: MFCD00237230) was used, with a molecular weight of 30,000–70,000 Da, for applications in biotechnology and cell culture. For this purpose, 0.05 mg of MXene was used to prepare the initial suspension in 1000 µL of DDW, which allowed a concentration of 5 × 10^2^ mg L^−1^. Then, 0.01 mg of poly-L-lysine was added to the suspension.

### 2.5. FTIR Measurements

Infrared spectroscopy (FTIR) measurements were made using a Perkin-Elmer System 2000 spectroscope. The device operates in the wavelength range of light from 370 to 4000 cm^−1^. During the analysis, the Diffuse Reflectance Infrared Fourier Transform (DRIFT) adapter was used, which enables the diffuse measuring technique. The main advantage of using this measuring technique is that the substance being measured does not have to be compressed to form a solid tablet and it is possible to measure e.g., powders or sediments. During DRIFT measurements, infrared radiation incident on the material being tested is subject to diffuse reflection and mirror reflection. This measuring technique is characterized by very high sensitivity, therefore a very small amount of substance is enough to obtain a reflection spectrum.

Qualitative analysis of the presence of poly-L-lysine on the surface of 2D flakes of the Ti_3_C_2_ MXene phase was performed by the DRIFT-FTIR method, with a Nicolet iS5 spectrometer (Thermo Scientific, Waltham, MA, USA). Samples were mixed with dried KBr at a concentration of 2.5 wt.%. The FTIR analyzer on which the tests were performed had a resolution of 2 cm^−1^. Spectra were recorded in the range of 4000–400 cm^−1^. The number of replicates performed for each measurement was 32. Thermo Fisher’s OMNIC software was used to process and analyze the obtained data (background correction). The following describes how to prepare samples before starting the analysis. To carry out the background measurement, 9 mg of pure, dried KBr was placed in a measuring cup and then analyzed. Subsequently, 3 mg of freeze-dried 2D Ti_3_C_2_, pure PLL or 2D Ti_3_C_2_/PLL were gently mixed with 9 mg KBr in a mortar for good homogenization of samples.

### 2.6. Antibacterial Properties Tests

An 8 mg sample of 2D Ti_3_C_2_ flakes was suspended in 1 mL ultrapure water (18 MΩ·cm) in a glass container. The suspension was treated in the ultrasound cleaner (35 KHz, 100 watt) for 20 min. At the same time, a solution of poly-L-lysine hydrobromide, (30,000–70,000 Da, Sigma-Aldrich, St. Louis, MO, USA) was prepared at a concentration of 8 mg mL^−1^. Then, 500 µL of the poly-L-lysine (PLL) solution was mixed with 500 µL of 2D Ti_3_C_2_ flakes and incubated for 30 min at a temperature of 25 °C. Then, the suspension of PLL and 2D Ti_3_C_2_ flakes was centrifuged at 15,000 rpm for 30 min. The supernatant was removed and ultrapure water was added to the precipitate. The procedure was repeated. Estimation of non-absorbed PLL-molecules was performed by high performance liquid chromatography equipped with size exclusion chromatography column BioSep-SEC-s2000 (7.8 × 300 mm) (Phenomenex, Torrance, CA, USA). The poly-L-lysine yield was detected at 220 nm using the reference sample (4 mg mL^−1^).

Subsequently, 10 µL of bacterial suspension were mixed with an appropriate volume (10 μL) of two-fold dilutions of 2D Ti_3_C_2_ flakes and placed into wells of a 96-wells microplate with non-transparent side walls (Eppendorf). Wells were filled with sterile ultrapure water up to the final volume of 100 µL. Wells containing an appropriate amount of bacterial cells without 2D Ti_3_C_2_ flakes were used as the negative control. The microplate was incubated at 25 °C for 6 h. After incubation time, the viability of bacteria was assessed using the drop plate assay [23].

### 2.7. Analysis of Cytotoxicity In Vitro

The cytotoxicities of pristine 2D Ti_3_C_2_ and Ti_3_C_2_/PLL flakes were tested on human skin malignant melanoma cells (A375, ATCC), and human immortalized keratinocytes (HaCaT, ThermoFisher, Waltham, MA, USA). The A375 and HaCaT cell lines were cultured under an atmosphere of 5% CO_2_ at 37 °C and 95% humidity using complete Dulbecco’s Modified Eagle’s Medium (DMEM, Sigma-Aldrich) with the addition of 10% (v/v) fetal bovine serum (FBS), 1% (v/v) of penicillin and streptomycin and 1% (v/v) of L-glutamine. Cellular survival was checked using tetrazolium dye 3-(4,5-dimethylthiazol-2-yl)-2,5-diphenyltetrazolium bromide (MTT) assay after 24 h of cells incubation with tested nanomaterials (0–500 mg L^−1^). Both cell lines were seeded at a density of 1 × 10^5^ cells per mL and kept overnight to allow their adhesion to the surface. Then, the media were replaced with a series of suspensions of examined MXenes (100 µL per well). The control groups were prepared in the absence of MXene. Each experiment was conducted three times independently. After the exposure time, the cells were washed twice using phosphate-buffered saline (PBS, Sigma-Aldrich, St. Louis, MO, USA) and then treated with MTT (Sigma-Aldrich, St. Louis, MO, USA) solution (0.5 mg mL^−1^ in PBS; 100 µL per well). The cells were incubated with MTT for the next 4 h, protected from light. The supernatant was then removed, and the formed violet formazan crystals were dissolved in dimethyl sulfoxide (DMSO, Sigma-Aldrich; 100 µL per well). The absorbance was measured at 570 nm. The cellular viabilities were expressed as a percentage compared to the control groups.

### 2.8. Cell Cycle Analysis

Determination of the cell cycle phases was performed in the presence of RNAse and propidium iodide (PI). The skin cells were seeded at a density of 105 cells per mL. The analysis was carried out after 24 h of exposure to the tested flakes at a specific concentration (i.e., 0 or 500 mg/L). Suspensions of flakes from above the cells were collected with a pipette, and then the cells were washed twice with 1 mL PBS. Then after collecting PBS, 1 mL of trypsin was added to the wells and incubated until the cells detached from the growth surface. Subsequently, the cell suspension was transferred to appropriate tubes and centrifuged at 1500 rpm for 5 min. The supernatant was then decanted and the pellets were suspended in 300 µL PBS. Then, 0.5 mL of frozen 70% ethanol was added to each tube and the samples were vortexed (Mo Bio Laboratories model Labnet VX100). They were recorded for 60 min in the ice bath. Subsequently 2 mL of PBS was added into tubes and centrifuged at 2000 rpm for 10 min. This procedure was repeated twice. Then, the supernatant was decanted and the pellets were suspended in 200 µL of PBS. Next, the mixture of 50 µL of RNAse and 11 µL of propidium iodide was added via pipette and samples were incubated for 30 min. at 37 °C, protected from light. Then, 1 mL of PBS was added to the tubes and centrifuged at 2000 rpm for 10 min. The cells suspended in 500 µL of PBS were filtered through 0,2 µm syringe filter and examined using a Beckman Coulter flow cytometer, model CytoFLEX A00-1-1102.

### 2.9. Statistical Manipulation

Statistical analysis of antimicrobial results obtained was carried out with STATISTICA 6.0 (StatSoft Inc., Tulsa, OK, USA) software. To estimate significance of difference Wilkinson paired tests were applied. Differences were considered significant at *p* < 0.05.

## 3. Results

### 3.1. Materials Characterization

**Figure 1:**
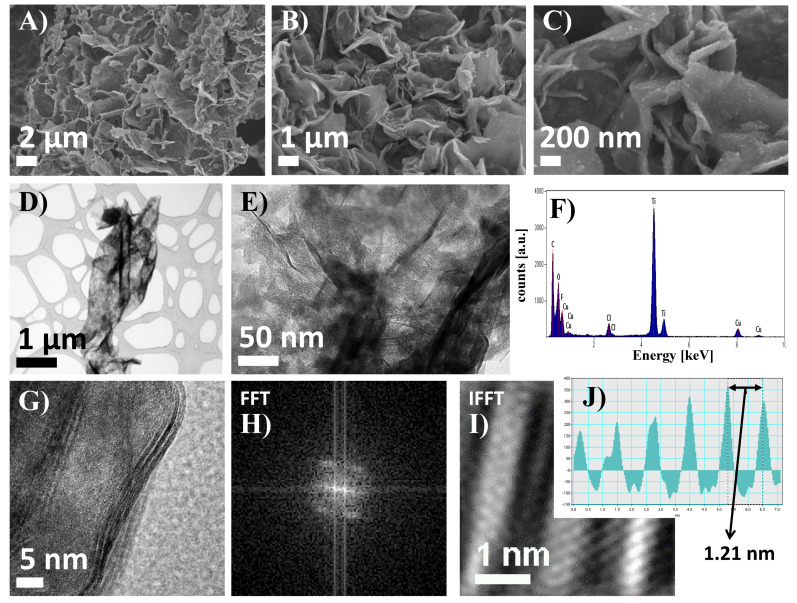
SEM images of 2D Ti_3_C_2_ MXene flakes taken at various magnifications (**A**–**C**); TEM images of a single 2D flake (**D**,**E**); results of corresponding EDS analysis (**F**), HRTEM image of flake edge (**G**), as well as fast Fourier Transform (FFT) (**H**), inverse fast Fourier Transform (IFFT) (**I**), images and resulting band intensity pattern (**J**).

Analysis of morphology and structure of 2D flakes of pristine Ti_3_C_2_ MXene after delamination was carried out using SEM and High-resolution transmissions electron microscopy (HRTEM), respectively. The results are presented in Figure 1. Morphology of 2D Ti_3_C_2_ MXene flakes was visualized at different magnifications (Figure 1A–C). The SEM images show friable agglomerates formed after the freeze-drying of the 2D Ti_3_C_2_ MXene flakes. Flakes have irregular shapes and sharp edges and are oriented differently towards the detector. For analysis of the 2D crystal structure, HRTEM was employed. The images taken at lower magnifications allowed for observation of a single MXene flake (Figure 1D,E) as well as the characteristic layered structure of differently oriented edges (Figure 1G). Layered reflection is also visible in the fast Fourier Transform (FFT) image (Figure 1H). In addition, the inverse fast Fourier Transform (IFFT) image shows alternating layers of light and dark bands (Figure 1I). It should be noted that each bright band corresponds to a single M_3×2_ layer i.e., Ti-C-Ti-C-Ti for the case of Ti_3_C_2_. On the other hand, each dark band corresponds to the ‘empty’ space between monolayers, formed after removal of A element from the MAX phase. To determine the characteristic distances between Ti_3_C_2_ monolayers in MXene flakes (light bands), band intensity pattern was taken based on IFFT image (Figure 1J). The measured distance between maximal intensities of two Ti_3_C_2_ monolayers is 1.21 nm and agrees with Ref. [24]. It is a characteristic widening of the distance in flakes after delamination i.e., a result of TMAOH intercalation and weakening of bonds between monolayers.

In order to determine the elemental composition of the flakes Energy-dispersive X-ray spectroscopy (EDS) analysis was performed (Figure 1F). The result of EDS analysis showed the elemental composition of the 2D flakes of the Ti_3_C_2_ MXene phase which is titanium, carbon, oxygen, fluorine, as well as copper and small amounts of chlorine. Titanium and carbon are elements included in the chemical composition of the tested MXene. The presence of copper probably comes from the copper mesh on which the sample was placed during the analysis. Elements such as oxygen and fluorine correspond to the functional groups present on the surface of the 2D flakes, which are formed as a result of HF-etching of A element [25,26]. The presence of chlorine could be the result of HCl usage for pH adjustment, according to the synthesis procedure.

**Figure 2:**
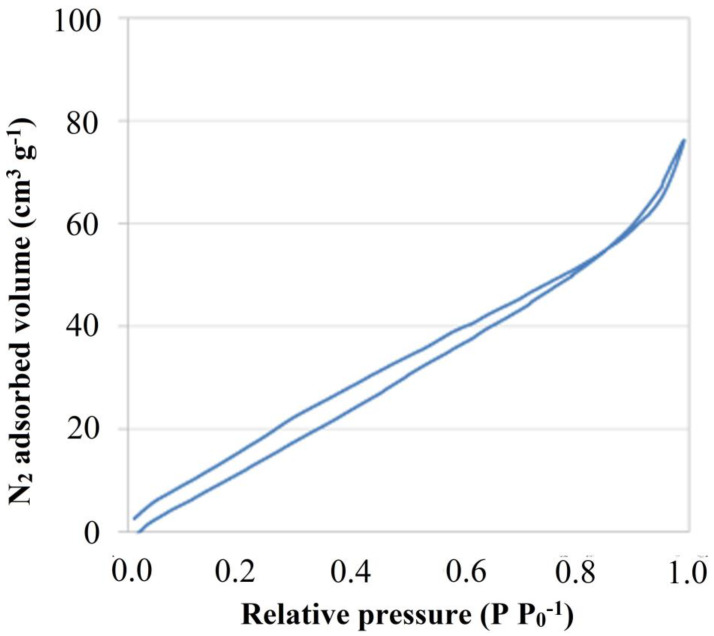
Isotherm of physical nitrogen sorption on the surface of 2D Ti_3_C_2_ MXene flakes.

The analysis of the physical properties of MXene flakes was performed based on experimentally determined N_2_ sorption isotherm. Comparing the obtained isotherm (Figure 2), together with those classified by the IUPAC, one can note that it resembles both the H3 and H4 type isotherms, corresponding to mixed-shape pores i.e., capillary and slit-shaped pores. The graph also shows a small hysteresis loop at higher relative pressures corresponding to the occurrence of capillary condensation in the slit pores. Based on the obtained isotherm, a specific surface area and porosity analysis for 2D Ti_3_C_2_ MXene were performed. Research carried out by A. Rozmysłowska-Wojciechowska et al. [17] showed that the specific surface area of etched Ti_3_C_2_ MXene can be c.a., 18 m^2^ g^−1^. Here, the obtained value of the total specific surface area of the 2D flakes after delamination and freeze-drying is 93.08 m^2^ g^−1^, the external surface area is 61.56 m^2^ g^−1^ and micropore surface area is 31.52 m^2^ g^−1^. It was also found that the surface area of micropores is about 34% of the total surface area. These values allow us to state that delaminated Ti_3_C_2_ MXene has about five times higher specific surface area compared to non-delaminated, only etched, multilayer systems.

### 3.2. Poly L-Lysine Adsorption Studies and Colloidal Stabilities

The subsequent tests involved analysis of PLL adsorption as well as verification of the stabilities of the obtained colloidal systems. Surface adsorption studies were carried out by using an experimental approach, similar to titration. At first, the colloidal system of pristine 2D Ti_3_C_2_ flakes was prepared in DDW. Then, poly-L-lysine (PLL) was added stepwise with a variable weight ratio of MXene:PLL from 1:0 up to 1:20.

**Figure 3:**
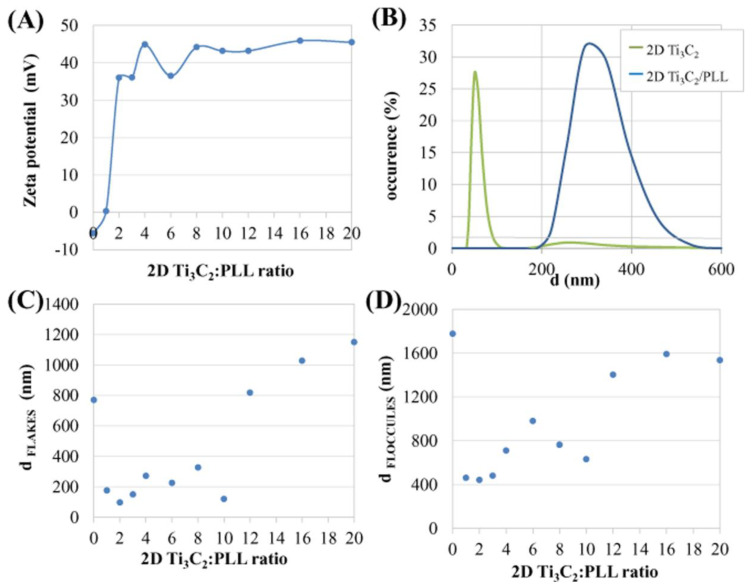
Changes of the zeta potential value of 2D Ti_3_C_2_ flakes as a result of gradual adsorption of poly L-lysine (**A**); Analysis of the hydrodynamic diameters of the 2D Ti_3_C_2_ MXene flakes after delamination and surface-modification by poly-L-lysine (PLL) (**B**); changes in the value of the hydrodynamic diameter of 2D Ti_3_C_2_ flakes (**C**), and floccules (**D**) as a result of gradual adsorption of poly L-lysine.

Results of the zeta potential changes for 2D Ti_3_C_2_ flakes as a result of gradual adsorption of PLL are shown in Figure 3A. Paying attention to the shape of the adsorption curve it can be seen, that adsorption of PLL changes the negative value of the zeta potential of the flakes to a positive one. The starting zeta potential value was −5.6 mV to reach the highest positive value of over +40 mV. As expected, a small addition of PLL to the MXene solution in a 1:1 proportion contributed to the most significant change of the surface charge of MXene. This indicates both the presence of very strong electrostatic interactions as well as the rapid and effective adsorption of the monolayer of PLL molecules on the surface of the MXene. The zeta potential in the subsequent stages increased linearly until the ratio reached 1:4. After exceeding this value, the zeta potential oscillated between +40 and +45 mV, which indicate multilayered surface coverage. It can be thus concluded that the two colloidal systems described above differ significantly in terms of zeta potential values. The studied colloid of 2D Ti_3_C_2_ flakes shows a negative value of zeta potential, i.e., −5.6 mV, while the colloid of 2D Ti_3_C_2_/PLL flakes has a highly positive zeta potential of 44.9 mV. The described analysis determines the way in which PLL affects the value of the zeta potential of 2D Ti_3_C_2_ flakes, and thus changes the ionic nature of the surface and finally—the surface electrostatic charge of the material. Research conducted by A. Rozmysłowska—Wojciechowska et al. [17] showed that the zeta potential for 2D Ti_3_C_2_ flakes after only HF-etching is −10.4 mV. Here, the zeta potential value is −5.6 mV. The difference in the results obtained is due to the probable surface-passivation (oxidation) of the flakes [27].

Parallel to zeta potential studies, analysis of the hydrodynamic diameters using the DLS method was carried out. Results for pristine 2D Ti_3_C_2_ and Ti_3_C_2_/PLL in a ratio 1:1 are presented in Figure 3B, whereas results for flakes and floccules during stepwise adsorption are presented in Figure 3C,D, respectively.

The distribution of the value of the hydrodynamic diameter of the 2D Ti_3_C_2_ and Ti_3_C_2_/PLL(1:1) flakes in DDW (Figure 3B) shows the presence of dispersed small flakes with hydrodynamic diameters of ~50 nm. For 2D Ti_3_C_2_/PLL flakes, the size of the hydrodynamic diameter was much larger (~350 nm). The above-described phenomenon of increasing the hydrodynamic diameter as a result of the addition of poly-L-lysine suggests the occurrence of flocculation. However, this effect is reversible after gentle manual shaking of the suspension which allows flakes to disperse again.

Figure 3C shows the change in the value of the hydrodynamic diameter of pristine 2D Ti_3_C_2_ flakes as a result of stepwise adsorption of PLL. After the addition of a small amount of PLL to the colloidal solution of MXene flakes in a ratio of 1:1, it was observed that the 2D Ti_3_C_2_ MXene flakes significantly reduce their hydrodynamic diameter from about 800–100 nm. This phenomenon corresponds to deflocculation and going into periodical stabilization. Along with increasing the concentration of PLL, until reaching the state where the ratio of MXene:PLL is 1:10, a fluctuation of the hydrodynamic diameters in the range from 100 nm to 300 nm was observed. This phenomenon is caused by a different alignment of 2D Ti_3_C_2_ MXene flakes towards the detector. However, it indicates the occurrence of a so-called plateau stage, where no changes are observed. After exceeding plateau, the hydrodynamic diameter increased rapidly, this allows deducing that 2D Ti_3_C_2_ MXene flakes are subjected to another stage of flocculation.

Hydrodynamic diameters of Ti_3_C_2_ floccules as a result of gradual adsorption of PLL are shown in Figure 3D. The values of the hydrodynamic diameters of flocculesrang from 400 nm to 1800 nm. In the first stage of increasing the concentration of PLL, similarly to Figure 3C, the phenomenon of deagglomeration occurs and the hydrodynamic diameters of floccules decreases. The hydrodynamic diameter increases to 1000 nm until the ratio of MXene:PLL reaches 1:7. This process demonstrates the attachment of individual 2D flakes to already formed floccules and their further enlargement. With the continuation of increasing the ratio of MXene:PLL, the hydrodynamic diameter of floccules decreases to 600 nm. After exceeding the value where the ratio of MXene:PLL is 1:10, the hydrodynamic diameter increases rapidly due to reaching complete flocculation.

A very important factor affecting the stability of 2D nanomaterials in colloidal systems is the pH of the environment [21]. Therefore, an analysis of the distribution of the hydrodynamic diameter values and the zeta potential was carried out as a function of pH in DDW.

**Figure 4:**
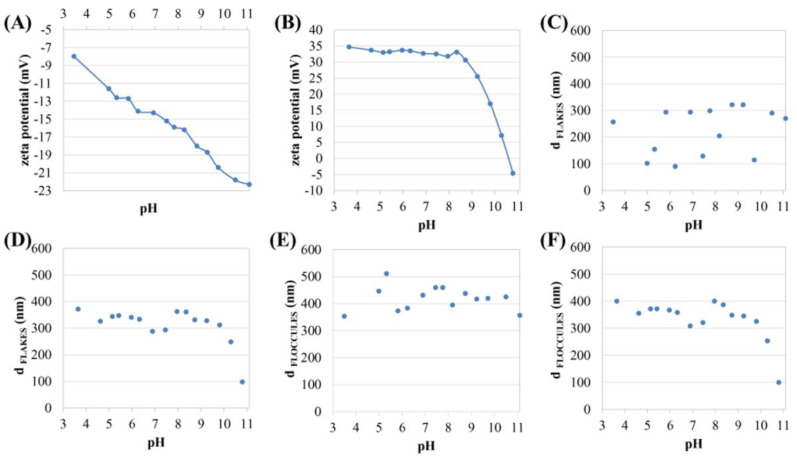
Distribution of the zeta potential values of 2D Ti_3_C_2_ (**A**), and 2D Ti_3_C_2_/PLL (**B**) flakes dispersed in double-distilled water (DDW) as a function of pH; distribution of the value of the hydrodynamic diameter of 2D Ti_3_C_2_ (**C**), and 2D Ti_3_C_2_/PLL (**D**) flakes dispersed in DDW as a function of pH; distribution of the value of the hydrodynamic diameter of 2D Ti_3_C_2_ (**E**), and 2D Ti_3_C_2_/PLL (**F**) floccules dispersed in DDW as a function of pH.

The distributions of zeta potential values obtained for 2D Ti_3_C_2_ flakes in DDW as a function of pH is shown in Figure 4A. When analyzing the zeta potential of 2D Ti_3_C_2_ flakes, it was observed that the stability of 2D Ti_3_C_2_ flakes increases linearly with pH. The zeta potential for 2D Ti_3_C_2_ flakes at pH ~ 4 was about −8 mV, while for pH ~ 11 c.a., −22 mV.

The distribution of the zeta potential value of 2D Ti_3_C_2_/PLL flakes in DDW as a function of pH is shown in Figure 4B. The analysis clearly illustrates the effect of the pH of the aqueous solution on the zeta potential of the 2D Ti_3_C_2_/PLL flakes. PLL changes the nature of the electric charge adsorbed on the surface of ion flakes from highly negative to highly positive. From a pH of about 3.5–8.5, a slow decrease in the zeta potential value can be seen. At pH ~ 9, the surface charge rapidly changes, passing through the isoelectric point at a pH of about 10.6 and returning to a negative value, as in the previous work on the adsorption of lysozyme on the surface of 2D Ti_3_C_2_ flakes [17]. It should be noted that a negative charge characterizes a clean flake surface. Thus, it can be concluded that PLL has been subsequently desorbed from the surface tested. The above result indicates the process of release of PLL from the surface of 2D flakes, along with an increase in pH, may be useful in drug delivery systems. After the PLL desorption process, the zeta potential for 2D MXene flakes is c.a., −5 mV, at a pH value of ~10.8, which however indicates incomplete removal from the MXene surface (clean surface value is ~22 mV). This may be due to strong electrostatic interactions between MXene and PLL.

Distribution of the values of the hydrodynamic diameter of 2D Ti_3_C_2_ flakes in DDW as a function of pH is shown in Figure 4C. It ranged from c.a., 100 nm up to 320 nm. Such large fluctuations are caused by the fact that the flakes dispersed in the solution position themselves in- or out-of-plane towards the detector. It should also be noted that 2D Ti_3_C_2_ MXene flakes are characterized by very small thicknesses, compared to lateral dimensions. Thus, the observed fluctuations are a characteristic feature of flake systems studied by the DLS method [17]. At the same time, the results obtained clearly show that there is no visible upward or downward trend for the determined range of fluctuations. In this aspect, one can, therefore, assume the relative stability of the colloidal system.

Figure 4D shows the distribution of the value of the hydrodynamic diameter of 2D Ti_3_C_2_/PLL flakes in DDW as a function of pH. The hydrodynamic diameters are in the range of ~300–370 nm from a pH value of about 3.5 to 9. However, after exceeding pH ~ 10 the hydrodynamic diameter drops sharply. During the release of PLL from the surface of 2D Ti_3_C_2_/PLL MXene flakes, along with the increase in pH, the stability of the colloidal system increases and at high pH values (alkaline environment) a decrease of the hydrodynamic diameter to at size of about 100 nm is observed.

The distribution of the hydrodynamic diameter value of the floccules formed by 2D Ti_3_C_2_ flakes in DDW as a function of pH is shown in Figure 4E. At pH values from ~3.5 to ~5.5, the floccules’ hydrodynamic diameter increases from about 350 nm to approximately 500 nm. After exceeding pH 5.5, the hydrodynamic diameter decreases and stabilizes at a value of ~400 nm. Small fluctuations in this value are also visible, but not as noticeable as in the case of individual flakes (Figure 4C). Therefore, it can be concluded that the formed floccules may have to some extent spheroidal dimensions.

The distribution of the hydrodynamic diameter value of floccules formed by 2D Ti_3_C_2_/PLL flakes in DDW as a function of pH is illustrated in Figure 4F. The sizes of floccules ranged from 300 nm to 400 nm. Similarly to Figure 4D, it can be seen that after exceeding pH ~10, the hydrodynamic diameter drops sharply.

The above-mentioned results reveal that the adsorption of PLL on the surface of 2D Ti_3_C_2_ flakes, by changing the surface sign, contributes to the formation of floccules. However, as the pH increases, it begins to desorb from the surface of 2D Ti_3_C_2_ flakes and finally, the stability of the colloidal system increases, and at high pH values (alkaline environment) deflocculation occurs. As can be seen, in the case of the tested MXene/PLL hybrids, the analysis made for both flakes and agglomerates indicates an almost identical course of curves (the differences are only minor), in contrast to pristine MXene. This means that the presence of PLL on the surface of the flakes, despite inducing a reversible flocculation process, allows for the unification of colloidal system features such as the presence of flakes and agglomerates (the number of agglomerates disappears in favor of single flakes).

### 3.3. FTIR Results

**Figure 5:**
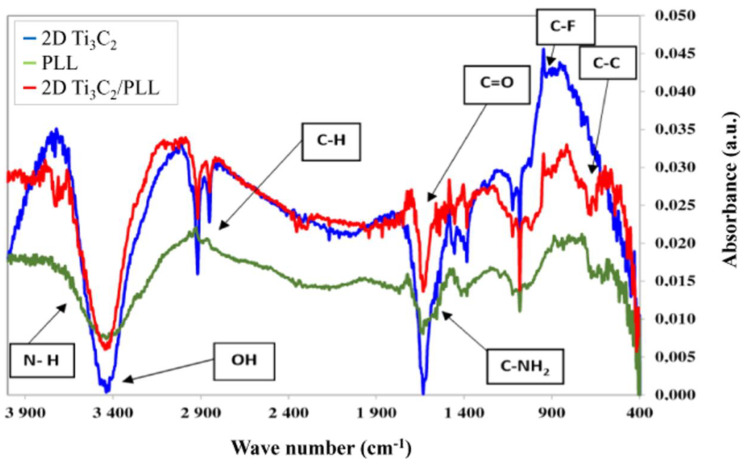
Comparison of FTIR analysis results for pristine 2D Ti_3_C_2_ MXene flakes, pure poly-L-lysine (PLL), and 2D Ti_3_C_2_ flakes surface-modified with PLL in a ratio of 1:1.

To confirm the presence of PLL on the surface of 2D Ti_3_C_2_ MXene flakes, pure PLL, pristine 2D Ti_3_C_2_ flakes as well as those modified with 1:1 ratio of PPL were investigated using Fourier-transform Infrared Spectroscopy (FTIR). The spectra obtained for these three samples are shown in Figure 5. The characteristic signals derived from bond vibrations have been marked on the obtained FTIR spectra. The spectrum obtained for PLL is consistent with the literature data [23,28]. In addition, in the case of 2D flakes of the MXene phase, the spectrum obtained is similar to that presented in the literature [24,29]. However, the spectrum obtained for 2D flakes of the Ti_3_C_2_ surface modified by PLL (2D Ti_3_C_2_/PLL) clearly indicates the presence of a mixture of signals from pure compounds. This is evidenced by the presence of signals from such functional groups as C-C at 815 cm^−1^, C-NH_2_ at 1546 cm^−1^ or N-H at 3638 cm^−1^, which occur in the case of pure PLL, and C-F at 947 cm^−1^ and C-H at 2879 cm^−1^ that occur in the pure phase of MXenes. In addition, a signal from the C=O group at 1675 cm^−1^ can be observed, which occurs in both tested materials. We have also successfully used an analogous analysis using FTIR to confirm the presence of collagen on the surface of MXene phases [30], therefore it can be concluded thatthe interactions are clearly electrostatically-driven. There is no additional feature present in the 2D Ti_3_C_2_/PLL FTIR spectrum that could not be found in pristine 2D Ti_3_C_2_ or pure PLL. The 2D Ti_3_C_2_/PLL FTIR spectrum in Figure 5 additionally shows that both 2D Ti_3_C_2_ and PLL are intact in the Ti_3_C_2_/PLL mixture, i.e., neither 2D Ti_3_C_2_ nor PLL degrades.

### 3.4. Analysis of Antibacterial Properties

**Figure 6:**
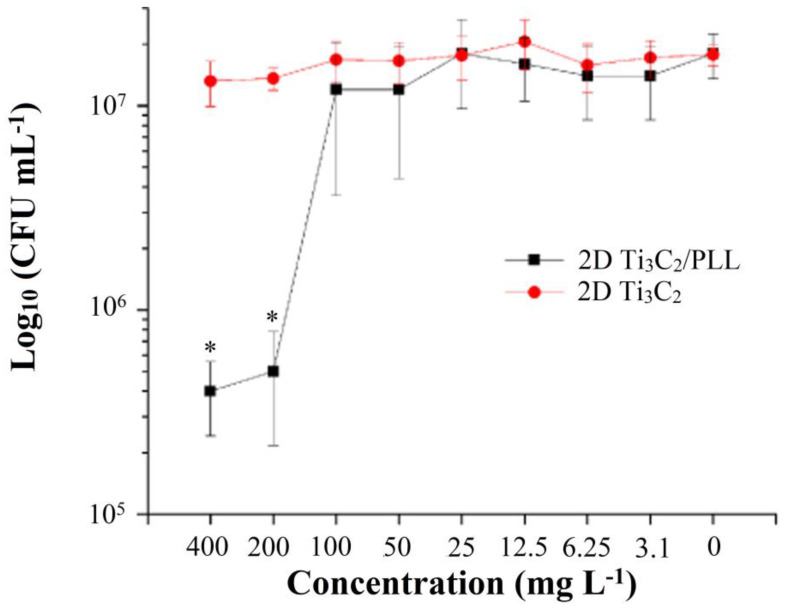
Evaluation of the bactericidal properties of pristine 2D Ti_3_C_2_ flakes as well as surface-modified with poly-L-lysine (PLL). Asterisk indicate significant differences at *P*  <  0.05.

In order to determine bactericidal properties, i.e., the number of colony-forming bacterial cells—Colony Forming Unit (CFU) in 1 mL solution, as a function of a decrease in the concentration of 2D Ti_3_C_2_ MXene phase flakes and 2D Ti_3_C_2_ flakes surface treated with poly-L-lysine, an analysis was performed, whose results are shown in Figure 6.

Unmodified 2D Ti_3_C_2_ flakes showed a lack of antimicrobial activity against Gram-negative bacterial cells of *E. coli* MG 1655. This result is expected, given the physicochemical properties of 2D Ti_3_C_2_ flakes. In particular, unmodified 2D Ti_3_C_2_ flakes tend to form aggregates in aqueous solution and have a negative charge (−15 mV, Figure 4), which does not contribute to antimicrobial activity upon contact with bacterial cells. As shown earlier, the pronounced antimicrobial properties of carbon nanoparticles are determined by direct contact with the surface of bacteria [31]. Positive charge and the size of the particle aggregates are the two factors that should contribute to antimicrobial activity [32].

Modification of 2D Ti_3_C_2_ flakes with poly-L-lysine made it possible to reduce the negative charge on 2D-flake’s surface, as well as to reduce its flocculation degree in aqueous solution. However, PLL is a well-known antimicrobial substance [19,33]. This fact determines the necessity of getting rid of unbounded PLL. Simple washing of 2D Ti_3_C_2_ flakes by precipitation and resuspension was enough to get rid of the unbound polymer, which was confirmed by the results of chromatographic analysis (Figure S1).

At the same time, 2D Ti_3_C_2_ flakes which were modified with PLL have been shown to possess some bactericidal properties. Thus, the co-incubation of *E. coli* cells with 2D Ti_3_C_2_/PLL flakes for 6 h reduced the number of living cells by two orders of magnitude at a concentration of 200 mg L^−1^ (Figure 6). This is a significant value (*p* < 0.05), whereas below this concentration a decrease in the number of viable E. coli cells was not observed. However, we could not record the complete elimination of living bacteria even at concentrations higher than 200 mg L^−1^.

The antimicrobial action of nanostructured carbon materials is realized through several mechanisms. One of them is a disruption of membrane functionality through respiratory chain inhibition [34], inhibition of bacterial energy metabolism [31] and oxidative membrane stress [35]. Such a mechanism underlies the action of fullerenes [36], graphene and its derivatives [35].

Another scenario involves direct puncturing [37] or cutting [38] bacterial cell walls and the underlying cytoplasmic membrane by the sharp parts of carbon-based nanomaterials.

Computer simulation showed that graphene nanomaterials can damage phospholipid bilayer by physical contact, however, the process more resembles the tearing of phospholipid molecules from the bilayer [39]. It should be noted, to fracture a bacterial cell upon contact with the nanostructured surface, it is necessary to apply significant stretching force, which is dependent on hydrophobicity/hydrophilicity of the surface, contact area and other [40].

As for the mechanism, which can describe the antimicrobial action of 2D Ti_3_C_2_, now we know that direct contact between cells and MXene is necessary, that has been provided by its appropriated modification. Further efforts from the science community are needed to understand the molecular basis of MXenes antimicrobial properties.

### 3.5. Analysis of Potential Cytotoxicity

**Figure 7:**
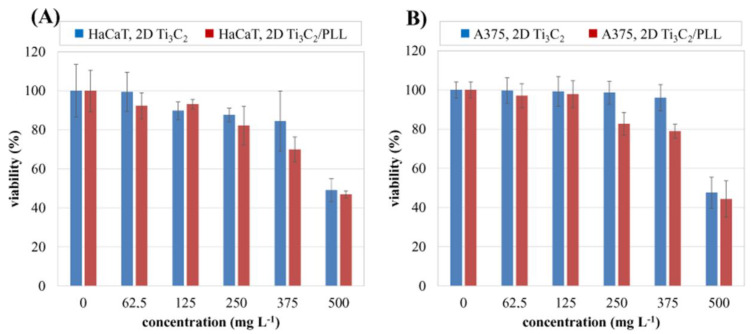
Relationship between the concentration of pristine 2D Ti_3_C_2_ and 2D Ti3C_2_/PLL flakes and the viability of normal skin cells (fibroblasts) of the HaCaT line (**A**) and malignant A375 cells (**B**) after 24 h of exposure.

In vitro cytotoxicity analysis, performed using the MTT test, allowed us to determine the relationship between the concentration of 2D Ti_3_C_2_ and 2D Ti_3_C_2_/PLL flakes and the viability of normal skin cells (keratinocytes, HaCaT) as well as malignant melanoma A375 cells. The test was performed after 24 h of incubation. The results of the analysis are illustrated in Figure 7. Obtained results indicate that surface modification with PLL does not significantly affect the cytotoxicity of the 2D flakes in the tested concentration range. The tested nanomaterial in both unmodified and surface-modified form has a similar effect on normal (Figure 7A) skin as well as malignant (Figure 7B) cell cultures. In the concentration range up to 375 mg L^−1^, it does not show cytotoxicity.

**Figure 8:**
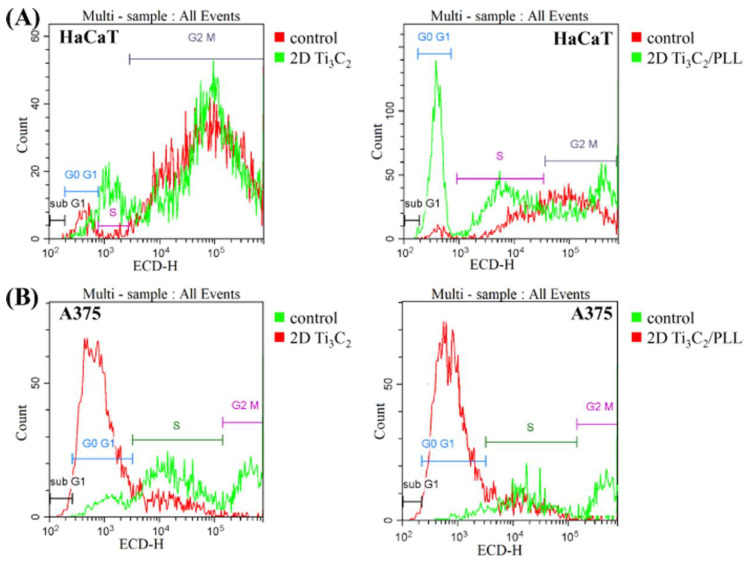
The influence of 2D Ti_3_C_2_ and Ti_3_C_2_ modified with poly-L-lysine (PLL) on cellular cycle in (**A**) HaCaT and (**B**) A375 cells.

To propose a potential mechanism of 2D Ti_3_C_2_ cytotoxicity (in both Pristine and PLL modified forms), we tested their influence on DNA synthesis after exposure to the highest applied concentration, which was toxic towards both skin cell lines. For such a purpose, cytometric analysis of the cell cycle after staining with propidium iodide was carried out. The obtained results are shown in Figure 8. After 24 h of incubation of pristine Ti_3_C_2_ with HaCaT cells, no meaningful differences in the cellular cycle were observed, compared to untreated controls. Interestingly, the coating of nanomaterial with PLL resulted in G0/G1 phase arrest, which suggests the occurrence apoptosis occurrence. A similar tendency (apoptosis triggering) was also noted in the case of melanoma cells treated with both types of tested nanomaterials. The dysfunction of proliferative potential and apoptotic cell death is proposed as one of the most desired mechanisms of action in anticancer treatment [41].

## 4. Conclusions

In this work, the effect of surface modification of two-dimensional (2D) flakes of the Ti_3_C_2_ MXene with poly-L-lysine (PLL) on the colloidal and biological properties of the developed materials was investigated. The developed 2D Ti_3_C_2_/PLL hybrids exhibited interesting bioactive properties e.g., bactericidal properties and good biocompatibility in a relatively wide concentration range (up to 375 mg/L). It was shown that PLL changes the nature of the electric charge present on the surface 2D MXene flakes. The adsorption occurred through electrostatic interactions. The tested 2D Ti_3_C_2_ flakes in DDW showed a negative zeta potential value of −5.6 mV, while 2D Ti_3_C_2_/PLL had a highly positive zeta potential value of +44.9 mV. The stability of 2D Ti_3_C_2_ increased linearly with increasing pH. The PLL, after exceeding pH ~10.8, probably desorbed from the surface of the Ti_3_C_2_ MXene flakes. The PLL adsorption allows obtaining better dispersion in DDW.

Stepwise adsorption of PLL contributes to a positive deflocculation of 2D flakes, which allows better dispersion of the nanomaterial in aqueous solution. Unmodified 2D flakes showed a lack of antimicrobial activity whereas the 2D Ti_3_C_2_/PLL hybrid possesses higher bactericidal properties against Gram-negative *E. coli*.

Incubation of *E. coli* cells with Ti_3_C_2_/PLL for 6 h reduced the number of viable cells by two orders of magnitude at 2–10 mg L^−1^. In the concentration range up to 250 mg L^−1^, the tested Ti_3_C_2_ MXene phase in both unmodified and surface-modified form did not show cytotoxicity.

## Supplementary Materials

The following are available online at https://www.mdpi.com/1996-1944/13/10/2347/s1, Figure S1: Determination of trace amounts of poly-L-lysine in the 2D Ti_3_C_2_ flakes sample using SEC-HPLC. 1—yield of the reference sample of poly-L-lysine (4 mg mL^−1^). 2—separation profile of the washed PLL-modified 2D Ti_3_C_2_ flakes.
