# Supplementary material for: Engineering of 2D Ti3C2 MXene Surface Charge and its Influence on Biological Properties
Source: Materials (Basel). 2020 May 20;13(10):2347. doi: 10.3390/ma13102347 (PMC7287753; doi:10.3390/ma13102347)
Supplement: Supplementary file 1 [file materials-13-02347-s001.pdf]

materials

Supplementary material

Engineering of 2D Ti_3C_2 MXene Surface Charge and its Influence on Biological Properties

Anita Rozmysłowska-Wojciechowska [1,*], Joanna Mitrzak [1], Aleksandra Szuplewska [2], Michał Chudy [2], Jarosław Woźniak [1], Mateusz Petrus [1], Tomasz Wojciechowski [2], Alexey S. Vasilchenko [3] and Agnieszka M. Jastrzębska [1]

1 Faculty of Materials Science and Engineering, Warsaw University of Technology, Wołoska 141, 02-507 Warsaw, Poland; asiamitrzak@gmail.com (J.M.); jaroslaw.wozniak@pw.edu.pl (J.W.); Mateusz.Petrus.dokt@pw.edu.pl (M.P.); agnieszka.jastrzebska@pw.edu.pl (A.M.J.)
2 Faculty of Chemistry, Warsaw University of Technology, Noakowskiego 3, 00-664 Warsaw, Poland; aszuplewska@ch.pw.edu.pl (A.S.); chudziak@ch.pw.edu.pl (M.C.); wojciechowski16@gmail.com (T.W.)
3 Institute of Environmental and Agricultural Biology (X-BIO), Tyumen State University, 625003 Tyumen, Russia; a.s.vasilchenko@utmn.ru
* Correspondence: anita.rozmyslowska@gmail.com

Received: 25 March 2020; Accepted: 14 May 2020; Published: date

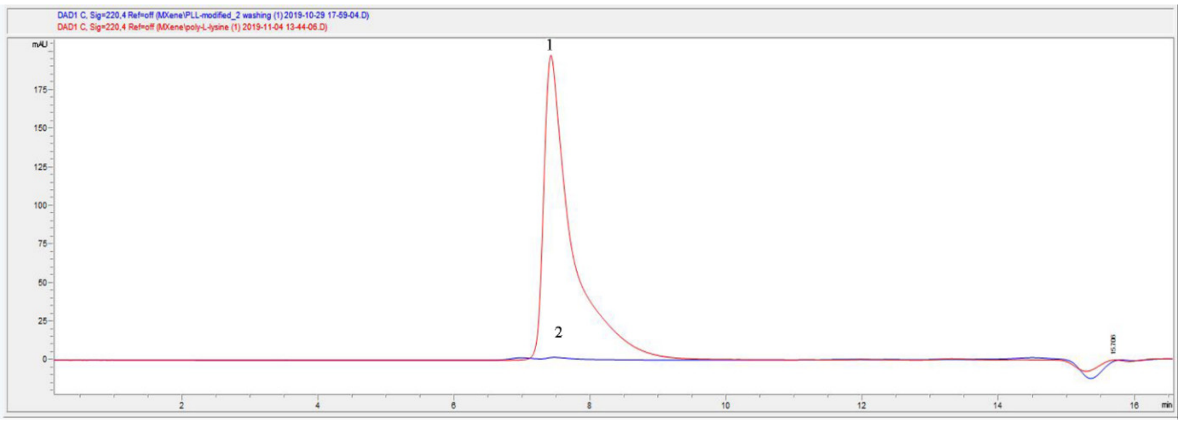

Figure S1. Determination of trace amounts of poly-L-lysine in the 2D Ti_3C_2 flakes sample using SEC-HPLC. 1—yield of the reference sample of poly-L-lysine (4 mg mL^{-1}). 2—separation profile of the washed PLL-modified 2D Ti_3C_2 flakes.

© 2020 by the authors. Submitted for possible open access publication under the terms and conditions of the Creative Commons Attribution (CC BY) license (http://creativecommons.org/licenses/by/4.0/).
